# The CICM second part viva reliability coefficient: Which index, for which decision, against which benchmark?^☆^

**DOI:** 10.1016/j.ccrj.2026.100209

**Published:** 2026-08-26

**Authors:** Shailesh Bihari, Sara Jane Allen, Michaela Jane Cartner, Sebastian Knudsen, Danielle Austin, Sarah Wesley

**Affiliations:** Department of Intensive and Critical Care Medicine, Flinders Medical Centre, College of Medicine and Public Health, Flinders University, Adelaide, South Australia, Australia; Cardiovascular Intensive Care Unit, Te Toka Tumai Auckland, Auckland, New Zealand; Intensive Care Unit, Gold Coast University Hospital, Southport, John Flynn Private Hospital, Tugun, Queensland, Australia; Intensive Care Unit, Royal Perth Hospital, Perth, Western Australia, Australia; Intensive Care, South Western Sydney Local Health District, Sydney, New South Wales, Australia; Intensive Care Unit, Royal North Shore Hospital, Sydney, New South Wales, Australia

To the Editor,

Hoffman et al. are to be commended for a transparent generalisability-theory analysis of 2774 individually scored viva stations.^1^ Their headline finding — a second part viva reliability of 0.61 dominated by case specificity (51.5%) — accords with decades of clinical-competence research and correctly identifies sampling more cases, rather than refining rubrics, as the principal lever for improvement.^2^ However, four points bear on its interpretation and benchmarking.

First, the coefficient does not match the decision. The viva at present yields a criterion-referenced pass/fail decision at a fixed 50% score, yet the reported index measures score reproducibility against a universe of stations. Their Fig. 1 equation places the station and examiner main effects in the denominator — the signature of an index of dependability (Φ) rather than the relative coefficient (Eρ^2^).^3^ Because Φ ≤ Eρ,^2^ a dependability-type coefficient is benchmarked against the 0.8 threshold defined for the relative coefficient, and with comparators such as the Royal Australian College of General Practitioners (RACGP) Clinical Competency Exam (CCE) (0.71) and the objective structured clinical examination literature (Cronbach's α 0.43–0.93) — that report relative coefficients or α, itself a lower bound that underestimates reliability, it is being compared with better-suited alternatives.[4], [5], [6] For a fixed pass mark (50%) with a 35% failure rate, the decision-relevant statistics are dependability at that mark (Φλ) and classification accuracy and consistency — how many candidates would receive the same result on retesting.^7^

Second, the examiner-stringency estimate of 1.4% is not credible as a true examiner effect. Examiners scored independently but could discuss and revise their scores before submission. The 0.91 inter-examiner correlation and the 1.4% component reflect post-hoc convergence within a nested, disconnected ‘circuits’ design in which examiner stringency and candidate ability are confounded — as the senior author's account of disconnected designs makes clear.^8^ Although conferral may dampen variance within an examiner pair, but stringency differences between pairs persist and are invisible in this design. In the structurally identical Membership of the Royal Colleges of Physicians of the United Kingdom (MRCP) (UK) Practical Assessment of Clinical Examination Skills (PACES), where examiners mark independently, examiner leniency–stringency contributed ∼12% of main-effect variance;^9^ the corresponding College of Intensive Care Medicine (CICM) share of main-effect variance is about 3.5%. A several-fold smaller component is more plausibly an artefact than a feature, and implies that 0.61 is optimistic. A sensitivity analysis on first-recorded (prediscussion) scores would help.

Third, no uncertainty is reported. Variance components — particularly the dominant candidate × station term — carry large sampling error. Point estimates without confidence intervals overstate precision.^3^ The 20-station projection extrapolates well beyond the observed eight and assumes exchangeable additions.

Fourth, appending the two Hot Cases as exchangeable viva stations is not composite reliability: these components are of different format, duration, and reliability (the Hot Case reliability is 0.42^10^) and require stratified, covariance-weighted estimation, and the reported rise to 0.64 may not survive it.

The 2019–2023 data predate subsequent viva changes, so these estimates may understate current performance. Borderline regression — the authors’ proposed remedy for station difficulty — has been piloted successfully in both the first part and second part (adult) examinations, with global ratings recorded alongside marks; the first part adopted it formally from the 2025.1 sitting, and the college has committed to its formal adoption for the second part, with guidance from the Australian Council for Educational Research, after appropriate notification to candidates.

None of this overturns the authors’ conclusion, which we share: the viva is imperfect, additional sampling is one potential remedy, borderline regression is likely to substantially improve performance, and programmatic assessment for the CICM training programme in its entirety merits consideration. Our point is narrower — the size of the deficit, the negligible examiner effect, the epoch of results sampled, and the cross-college comparison are each shaped by analytic choices that should be made explicit, so that the credentialling decisions resting on them are demonstrably defensible.

## Funding

This work received no specific funding.

## Author Contribution

All authors contributed equally and approve the manuscript.

## Conflict of interest

All authors are current examiners for the College of Intensive Care Medicine Second Part examination. Sara Jane Allen is the Chair, and Michaela Jane Cartner the immediate past Chair, of the Second Part Examination, College of Intensive Care Medicine of Australia and New Zealand.
